# Olive Mill Wastewater Fermented with Microbial Pools as a New Potential Functional Beverage

**DOI:** 10.3390/molecules28020646

**Published:** 2023-01-08

**Authors:** Paola Foti, Paride S. Occhipinti, Nunziatina Russo, Antonio Scilimati, Morena Miciaccia, Cinzia Caggia, Maria Grazia Perrone, Cinzia L. Randazzo, Flora V. Romeo

**Affiliations:** 1 Department of Agriculture, Food and Environment (Di3 A), University of Catania, Via Santa Sofia 100, 95123 Catania, Italy; 2 ProBioEtna srl, Spin-Off of University of Catania, Via Santa Sofia 100, 95123 Catania, Italy; 3 Department of Pharmacy-Pharmaceutical Sciences, University of Bari “Aldo Moro”, Via E. Orabona 4, 70125 Bari, Italy; 4 CERNUT (Interdepartmental Research Centre in Nutraceuticals and Health Products), University of Catania, Via le A. Doria 6, 95125 Catania, Italy; 5 Consiglio per la Ricerca in Agricoltura e l’Analisi dell’Economia Agraria (CREA), Centro di Ricerca Olivico-Tura, Frutticoltura e Agrumicoltura, Corso Savoia 190, 95024 Acireale, Italy

**Keywords:** olive mill wastewater, microfiltration, fermentation, microbial pool, hydroxytyrosol, functional beverage

## Abstract

Olive mill wastewater (OMWW) represents a by–product but also a source of biologically active compounds, and their recycling is a relevant strategy to recover income and to reduce environmental impact. The objective of the present study was to obtain a new functional beverage with a health–promoting effect starting from OMWW. Fresh OMWW were pre–treated through filtration and/or microfiltration and subjected to fermentation using strains belonging to *Lactiplantibacillus plantarum*, *Candida boidinii* and *Wickerhamomyces anomalus*. During fermentation, phenolic content and hydroxytyrosol were monitored. Moreover, the biological assay of microfiltered fermented OMWW was detected versus tumor cell lines and as anti-inflammatory activity. The results showed that in microfiltered OMWW, fermentation was successfully conducted, with the lowest pH values reached after 21 days. In addition, in all fermented samples, an increase in phenol and organic acid contents was detected. Particularly, in samples fermented with *L. plantarum* and *C. boidinii* in single and combined cultures, the concentration of hydroxytyrosol reached values of 925.6, 902.5 and 903.5 mg/L, respectively. Moreover, biological assays highlighted that fermentation determines an increase in the antioxidant and anti–inflammatory activity of OMWW. Lastly, an increment in the active permeability on Caco-2 cell line was also revealed. In conclusion, results of the present study confirmed that the process applied here represents an effective strategy to achieve a new functional beverage.

## 1. Introduction

Olive oil by-products, while representing a management problem for olive oil companies, may actually represent a source of high value–added compounds that can be used in pharmaceuticals, food, feed and cosmetics for their health properties [1,2,3]. In fact, olive mill wastewater (OMWW) is a resource rich in phenols including hydroxytyrosol (HT) and tyrosol (TYR), characterized by high antioxidant, anti–inflammatory, antimicrobial and anticarcinogenic activities [4]. The scientific community has proposed several strategies for the valorization of this by–product including solvent extraction techniques, selective resins, membrane filtration or enzymatic applications [5]. These techniques allow the extraction and/or concentration of bioactive compounds in order to increase the nutraceutical component and produce new products or functional ingredients, thus, responding to the demand of consumers who are now aware of the beneficial role that these natural products play in human and animal diets. In the food industry, OMWW have been proposed as an added functional ingredient in meat, dairy, fish, bakery products and juices [6,7,8]. As a matter of fact, the addition of such phenolic components in food matrices has been shown not only to fulfil a technological function (i.e., to extend the shelf life) but also to improve the health and safety properties of the food. Although the interest of the scientific community in the use of microorganisms in the bioprocessing of agro-industrial waste has grown in recent years [9], only a few microbial applications have been proposed for the valorization of this matrix. Authors have reported that the use of live microorganisms increases the content and bioavailability of the phenolic compounds, and especially of HT and TYR [10,11]. In addition, the driven microbial fermentation provides several advantages by preserving and improving food safety and shelf life due to the formation of organic acids, such as lactic, acetic, formic, propionic acids, etc. [12]. The diversities of acids are dynamic among different alcoholic beverages and fermented food, as are the synergistic effects of abiotic and biotic factors [13]. Functional microorganisms, such as lactic acid bacteria (LAB) and yeasts, are responsible for the metabolism of organic acids. Therefore, the use of selected microorganisms, especially yeasts and LAB isolated from spontaneous similar fermented matrices such as table olives, could represent a low–cost strategy to stabilize and improve the nutraceutical and sensory traits of OMWW. In detail, *Lactiplantibacillus plantarum* strains from fermented olives have been largely associated with the metabolism of phenolic compounds as they can produce degradation enzymes, such as β-glucosidase, esterase, tannase, decarboxylase [14]. Moreover, some of them have been proposed due to their potential probiotic activity [15]. With regard to yeasts, several species show β-glucosidasic, lipasic and esterasic activity and have been used for their ability to improve sensorial profile through production of esters from fatty acids and free fatty acids. Among yeasts, *Candida boidinii* and *Wickerhamomyces anomalus* are the most commonly used as starters [16]. In addition, yeasts isolated from oil matrices, especially strains of *W. anomalus*, have demonstrated several probiotic characteristics, among which the most known is the in vitro cholesterol removal capacity [17].

Today, the functional beverage sector is steadily increasing worldwide thanks to its high nutritional value and the possibility to add flavors. Furthermore, nutraceutical beverages with added probiotics and prebiotics are of considerable interest to the consumer [18], as this matrix was shown to inhibit proliferation and induce apoptosis in several tumor cells, prevent DNA damage and exert anti–inflammatory activity [19].

The aim of this study was to set up a process to obtain a new functional beverage with a health–promoting effect starting from OMWW. For this purpose, OMWW were pre–treated through filtration and microfiltration and then subjected to fermentation with selected microbial pools, isolated from spontaneously fermented table olives. During fermentation, the biotechnological aptitude of the different strain combinations, their effect on the fermentation parameters, the increase of the phenolic content, especially as HT increase, were evaluated. Furthermore, a biological characterization to evaluate the safety profile and the antioxidant activity was performed on treated OMWW samples. Finally, the ability to cross Caco-2 cell monolayers, as a model of gastrointestinal tract absorption, was performed. 

## 2. Results

### 2.1. Chemico-Physical Characterization of Sample of Different Trials

The OMWW belonging to Trial I were monitored at different times (0, 8, and 30 days), through the detection of pH, total soluble solids (TSS), total phenol content and single phenols by HPLC (Table S1). Regarding pH, any significant difference was observed at the beginning of fermentation, and the lowest pH value (4.45) was reached at T8 in sample inoculated with *C. boidinii* in single culture. The TTS at the beginning of fermentation showed values between 7.08 and 8.32, reaching values between 5.60 and 6.34 at T30. During the fermentation, the total phenol content showed, to some extent, a constant trend, reaching the highest concentration at T30 in samples fermented with *W. anomalus* in single culture, with a value of 3241.9 mg/L. The results obtained by HPLC confirmed this increase, as samples treated with *W. anomalus* showed the highest concentration of HT, equal to 2630.4 mg/L. Regarding TYR, an increase during fermentation was observed, reaching, after 30 days, values between 508.6 and 679.4 mg/L in all treated samples. The chemical analyses performed on Trial I were repeated on Trial II (Table S1). The pH decreased during fermentation, showed the lowest values at T8. In detail, all inoculated samples showed a lower pH than the control sample. In particular, the lowest value was found in the samples with *L. plantarum* and *W. anomalus* in single culture, but also in the combination *L. plantarum* and *C. boidinii* and, finally, with the mix of the three strains with values ranging from 3.97 to 3.99. With regard to TSS, the greatest decrease occurred with the combination of *L. plantarum* in association with *W. anomalus,* going from a value of 7.84 at T0 down to 5.50 at T8. At T30, almost all samples maintained the value showed at T8 of fermentation. In addition, total phenols at T8 and T30, in all inoculated samples, showed a higher content over time compared with the control sample. In detail, the samples with significantly higher phenolic content were the three–strain association (3379.5 mg/L) and *W. anomalus* in single combination (3261.9 mg/L) at T8, while at T30 was the sample inoculated with *L. plantarum* with a value of 3577.6 mg/L. The results obtained by HPLC showed a decrease at T8 of HT in all samples except in the samples with *L. plantarum* where there was an increase of 115 mg/L of HT, and the sample inoculated with *L. plantarum* with *W. anomalus* which showed an increase equal to 262 mg/L of HT. All samples inoculated up to T30 had higher HT content. Opposingly, the TYR decreased during fermentation from an average range of values from 319.7 mg/L to 136 mg/L.

In Trial III, microfiltration resulted in a clear and sterile matrix. Before starting the final fermentation, a preliminary test was carried out in a reduced volume (100 mL) to ascertain if any difference could be revealed between trials with the addition of glucose, peptone and yeast extract (added at the same concentrations) and the trials without any additions. The results showed the same pH values and cell density during fermentation. Moreover, the addition of these compounds made the OMWW turbid (data not shown). For these reasons, to improve the acceptability of the product to consumers, the thesis without additions was chosen for the final test. During fermentation, pH, TSS and total phenol content were monitored (Table 1). Regarding pH, no significant difference was found at the beginning of fermentation. The pH at T0 was in a range of 5.12 and 5.19. Fermentation stopped at T21 for all samples examined. The end of fermentation was revealed by the stabilization of the pH value that was evaluated every three days of fermentation (data not shown). In particular, the samples inoculated with *W. anomalus* in single culture and in association with *L. plantarum* reached a pH value of 4.54 and 4.49 at T21, respectively. In addition, although slower than the previous theses, the theses containing *L. plantarum* and *C. boidinii* in single culture also reached at T21 a pH of 4.65 and 4.60, respectively. Total soluble solids showed no significant difference at any of the fermentation times. Initial values ranged from 8.30 to 10.85 °Brix, while values between 5.32 and 8.17 °Brix were reached at the end of fermentation. The sample used as a control during fermentation maintained its pH and TSS values. Regarding the content of total phenols, the highest values at the beginning of fermentation were found in the sample containing the *L. plantarum* and *W. anomalus* combination, a value that decreased during the fermentation process. In contrast, the sample with the three–strains combination showed an increase in total phenol content up to T14 with a value of 4015 mg/L, and then decreased at T21 reaching a value of 1543 mg/L.

### 2.2. Microbiological Analyses

Results on microbiological analyses (Table S2) are referred at the same sampling times reported for chemical analyses. Overall, for samples of Trial I, high microbial densities were detected for aerobic mesophilic bacteria, enterobacteria, and yeast in all sampling times. Regarding LAB, an increase of 1 Log unit at T8 was detected, and the values were quite constant until T30, with some exceptions. In detail, at the beginning of fermentation, the sample treated with *L. plantarum* showed a significantly higher cell density, with a value of 5.85 log CFU/mL, whilst at T30 the highest LAB densities were detected in samples treated with the combination of the three strains, namely of *L. plantarum* and *C. boidinii*, *W. anomalus* in samples inoculated with *W. anomalus* in single culture, and in samples treated with *L. plantarum* and *C. boidinii* in mixed cultures. Yeasts and molds also showed a similar trend in all samples. In fact, cell density increased at T8 of fermentation and then decreased at T30, when an average value of 6.45 Log CFU/mL were detected. Aerobic mesophilic bacteria counts showed only a slight variation during fermentation, reaching a final mean value of 6.25 Log CFU/mL, whereas Enterobacteriaceae and staphylococci showed a significant decrease during fermentation. At the beginning of fermentation, the latest microbial groups showed an initial average value of 6.63 and 3.05 Log CFU/mL, respectively. These values decreased significantly during fermentation in the inoculated samples, reaching values under the detection limit. In Trial II, the LAB and yeast counts increased during fermentation (Table S2). In detail, the LAB mean value starting from 4.27 Log CFU/mL reached, after 30 days, a mean value of 7.34 Log CFU/mL, whilst in samples inoculated with *W. anomalus* it reached the lowest cell density. A similar trend was observed for yeasts that at the 30th day exhibited a mean cell density of 9 Log CFU/mL in the sample inoculated with *L. plantarum* and *C. boidinii* in mixed culture. Aerobic mesophilic bacteria were found at high density, until the end of fermentation when a final average value of 7.52 Log CFU/mL was counted. Different trends were observed for Enterobacteriaceae and staphylococci, for which after a slight increase a significant decrease was detected after 30 days in all samples.

Regarding Trial III, before starting fermentation, the microfiltered OMWWs were subjected to microbiological analyses to confirm the achieved sterility. The following microbial groups were searched: LAB, yeasts, staphylococci, total mesophilic aerobic bacteria, Enterobacteriaceae and *Clostridium perfringens*. All used media and conditions are reported in Section 4. 

**Figure 1:**
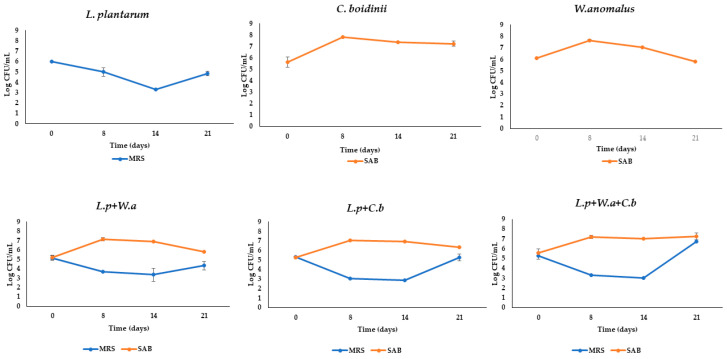
Microbial counts detected in MRS and SAB during fermentation in microfiltered OMWW differently inoculated. Data are expressed as means of Log CFU/mL ± standard deviations.

Once the OMWWs were analyzed, the selected strains were inoculated at a cell density of 10^8^ and 10^7^ CFU/mL for *L. plantarum* and yeasts, respectively. As shown in Figure 1, a different growth pattern between the two yeasts and the LAB strains was observed during fermentation. In fact, while in all inoculated samples LAB showed an initial decrease, during the first 14 days they increased until the 21st day; the yeasts increased their cell density during the first 18 days, when they reached values between 7.03 and 7.78 Log CFU/mL.

### 2.3. Phenol and Organic Acid Detection 

**Figure 2:**
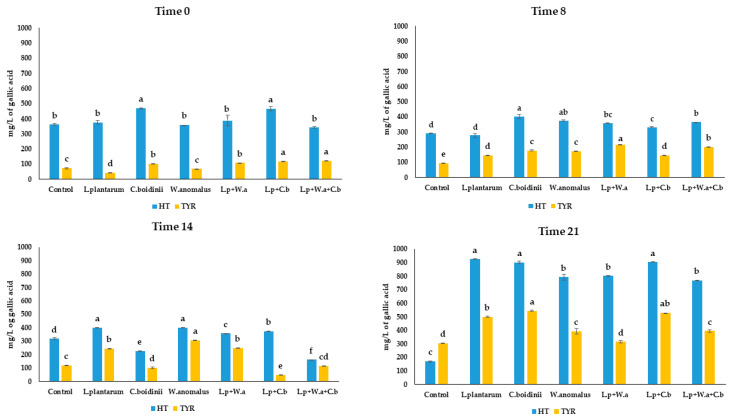
Concentration of HT and TYR during fermentation in microfiltered OMWW differently inoculated. Different letters indicate statistical differences within the columns for the same compound (significance at *p* ≤ 0.01).

Regarding phenolic content, HT and TYR were the main detected compounds, found at high concentration by HPLC during fermentation (Figure 2). As for HT, at the beginning of fermentation a concentration between 341.7 and 469.1 mg/L was found. At the end of fermentation, an exponential increase of HT in all inoculated samples was observed. Particularly in the samples treated with *L. plantarum* and *C. boidinii* in single and in combined cultures, the HT concentration was found as 925.6, 902.5 and 903.5 mg/L, respectively. A slowly increase in concentration of TYR was observed during fermentation, reaching values between 315.6 and 544.7 mg/L in all inoculated samples. In contrast, the control samples showed a significant decrease in HT along fermentation, reaching values of 170.6 mg/L and a slight increase in TYR, reaching final value of 303.7 mg/L.

In addition, organic acids were evaluated at the end of fermentation in all samples. The control sample, at the beginning of fermentation, was used as an initial control (Table 2). The control, analyzed at both the beginning and at the end of fermentation, showed a constant value of acids except for butyric, for which a concentration of 566.4 mg/L was detected only at T21. For all samples inoculated with the different microbial combinations, on the other hand, an acid increase during fermentation was observed, except for isobutyric acid that decreased in sample inoculated with *W. anomalus* and in all the inoculated combinations. In detail, the sample inoculated with *L. plantarum* showed the highest increase for all the detected acids.

### 2.4. Biological Assay

#### 2.4.1. Transepithelial Transport through Caco-2 Cell Monolayers

The intestinal permeability values, estimated with the Caco-2 cell experimental model, correlate well with human in vivo absorption data for many drugs and chemicals. Caco-2 cells are a human colon epithelial cancer cell line that, when cultured as a monolayer, differentiate to form tight junctions between cells to serve as a model of paracellular movement of compounds across the monolayer. The monolayer represents the human intestinal epithelial cell barrier and by this assay, the measured endpoint is intestinal permeability (expressed as apparent permeability—P_app_ value) (Table 3).

The flux from the apical part of the monolayer to the basolateral side (BA) is referred to the passive transport, while the measurement of active transport is obtained by measuring the reversed flow (AB), since Caco-2 cells express efflux pumps in the apical side. The smaller the BA/AB ratio value, the greater the contribution of the active transport to the membrane crossing. In all tested samples, the contribution of active transport to the membrane crossing was always lower than that due to passive diffusion, as demonstrated by high values of P_app_ AB. This occurs mainly for samples inoculated with *L. plantarum* and *C. boidinii*, which showed the highest value (as 1125 nm/s). This value could be related to a synergic effect between the LAB and the *C. boidinii* strains, that also in single cultures showed P_app_ values of 1014 and 575 nm/s, respectively. This result is confirmed by the lowest BA/AB value (2.58), detected in samples fermented with *L. plantarum* and *C. boidinii*. 

OMWW samples and HT pure (used as a control) at the opportune dilution (1:25) have been evaluated on different cell lines, normal (HepG2) and tumoral (Caco-2), in order to evaluate their toxicological profile [20]. Since no cytotoxic effect was detected (data not shown), they resulted to be safe at a dilution of 1:25, while with higher concentrations (as such and 1:10) a cytotoxic effect was registered. These results are in agreement with data reported by Di Mauro et al. [21], confirming that the use of higher concentrations (as such and 1:10) induced a reduction in cell viability in a dose–dependent manner, while lower concentrations did not affect cell viability.

#### 2.4.2. Evaluation Activity on COX-1 and COX-2 Isoenzymes

COX, also called Prostaglandin H synthase (PGHS), is a key enzyme in the inflammatory cascade. It catalyzes the conversion of arachidonic acid (AA) in prostanoids, bioactive lipids mediating numerous physiological and pathological processes in the body. Prostanoids include thromboxane A2 (TXA2), prostaglandins (PGD2, PGE2, PGF2α) and prostacyclin (PGI2). Two COX isoforms are known, COX-1 and COX-2, encoded by different genes. The two isoforms show 60% homology in their amino acid sequence. COX-1 is the isoform constitutively expressed in most tissues and responsible for maintaining normal physiological functions such as gastric protection, modulation of platelet function, and renal homeostasis. COX-2, differently from COX-1, is the inducible isoform upon pro–inflammatory stimuli. The possibility of finding anti–inflammatory properties in nutraceutical compounds would make the products under study extremely interesting, thus, the OMWW fermented sample inhibition of *ovine*COX-1 (*o*COX-1) and *human*COX-2 (*h*COX-2) enzyme activity was investigated and HT was used as positive control. Pure HT showed, at a concentration of 40 mg/L, inhibition activity on *o*COX-1 and *h*COX- 2 with a percentage of 6.41 and 26.11, respectively (Figure S1). The control OMWW sample did not show any anti–inflammatory activity, while low anti–inflammatory activity was found for the different OMWW samples. In detail, samples fermented with *L. plantarum*, *C. boidinii* and *W. anomalus* in single culture showed a moderate inhibitory activity towards both isoforms (Table S3). In particular, the sample inoculated with *L. plantarum* in single culture showed a selective inhibition of *o*COX-1, whereas samples treated with *C. boidinii* showed an inhibition towards both *o*COX-1 and *h*COX-2 with the percentage of inhibition reaching 15.96% and 12.95%, respectively. In addition, the sample inoculated with a combo of *L. plantarum* and *C. boidinii* preserves a selective inhibition towards *o*COX-1, with an inhibition of 8.20%. It could be hypothesized that *C. boidinii* produces some metabolites with a greater affinity and selectivity towards *o*COX-1 isoform. 

#### 2.4.3. Antioxidant Activity

**Figure 3:**
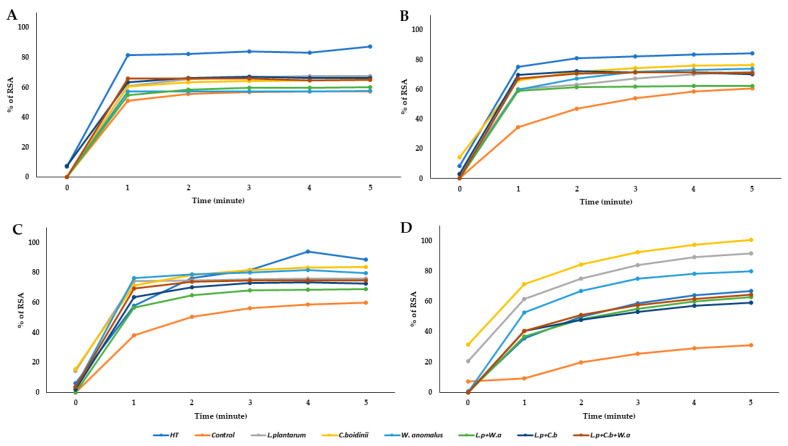
Evaluation of antioxidant activity expressed as % RSA. Each graph corresponds to a volume (µL) used for each sample: (**A**) 50 µL of samples; (**B**) 37.50 µL of samples; (**C**) 25 µL of samples; (**D**) 12.5 µL of samples.

Diabetes, cardiovascular diseases, arthritis and joint diseases, allergies and chronic obstructive pulmonary diseases are classified, according to the World Health Organization, as specific inflammation–mediated chronic diseases. The processes underlying these diseases are many, but oxidative stress is undoubtedly involved in their pathogenesis and in the development and establishment of a sustained inflammatory state. All selected samples were evaluated for their antioxidant activity by measuring their reactivity with 1,1-diphenyl-2-picrylidrazyl (DPPH), a purple–colored stable radical that strongly absorbs at λ = 517 nm, in order to determine their efficacy as scavengers of stable free radicals. Testing was carried out to compare the effect of fermented OMWW samples with the known antioxidant activity of HT (Figure 3). The data showed that the OMWW control exhibited lower antioxidant capacity, at all dilutions tested, compared with both that exerted by HT and fermented samples. In particular, at the lower tested volume (12.5 µL), the best antioxidant activity was obtained in the sample inoculated with *C. boidinii*, reaching a % RSA value higher than pure HT. The same behavior was observed for samples inoculated with *L. plantarum* and *W. anomalus,* in single culture.

## 3. Discussion

Fermentation is widely considered a low–cost strategy to recovery and valorize agro-industrial by–products [22]. In this study, in order to obtain a suitable matrix to be fermented with selected microbial pools, different Trials were set up. For this purpose, fresh OMWWs were collected at two successive seasonal years. Samples obtained from Trial I, untreated fresh OMWW, appeared very turbid and rich in unwanted solids at both the beginning and end of fermentation. Therefore, in Trial II, the OMWWs were subjected to on farm filtration using carton filters with different porosity. To date, such a technique is used to remove unwanted solid components from the matrix, maintaining the nutritional compounds as phenolic fraction (Figure S2) [23].

Results of Trial II showed that although the OMWWs (filtered through cardboard filters) visually appeared as clear from a physical point of view, at both the beginning and end of fermentation, they were not microbiologically suitable, in relation to the high total aerobic mesophilic bacteria densities. According to the European Regulation (EC) No. 1441/2007, the absence of pathogens, such as *Salmonella* spp. and *L. monocytogenes*, is considered an essential criterion for the microbiological safety of vegetable products, while no mandatory microbiological criterion is fixed for total aerobic mesophilic bacterial count. However, some guidelines include *Escherichia coli* and total aerobic mesophilic count as quality parameters, fixing the following thresholds (as CFU/g): *E. coli* < 10 for satisfactory; between 10 and ≤10^2^ for acceptable; and >10^2^ as not acceptable [24]. The same authors, for total aerobic mesophilic count, proposed the following thresholds: ≤10^4^ for satisfactory, between 10^4^ and lower or the same of 10^6^ for acceptable, and >10^6^ not acceptable, respectively [24]. Therefore, OMWW obtained through the last cardboard filter, with a porosity between 0.20 and 0.40 μm, were afterwards subjected to microfiltration (0.22 µm) in the laboratory. This procedure resulted in a microbiologically sterile, clear matrix mainly composed of phenols (Figure S3).

To date, the microfiltration technique is successfully applied in food industries, such as the dairy industry, as it induces an improvement in the microbial quality of the final product [25]. In the present study, the application of such a strategy allowed the evaluation of the biotechnological aptitude of the strains, used as single or mixed cultures, and enabled an understanding of how they interact with the matrix. The results showed that the use of microbial starters drove fermentation by lowering the pH to values as low as 4.49 and inducing an increase in the phenolic compounds. In detail, the combinations of *L. plantarum* and *C. boidinii,* both in single and in mixed cultures, resulted, at the end of fermentation, in the highest HT content, with values of 925.6, 902.5 and 903.5 mg/L, respectively. No oleuropein was detected at any sampling time as found by other authors [5,26]. Although *L. plantarum* is mainly known for its β-glucosidase activity or its probiotic potential [27], in all the tests carried out, there was a slight decrease of LAB count in sample with *L. plantarum* that showed an increase only after t14 of fermentation (Figure 1). This suggests that these strains are able to utilize certain metabolic pathways to survive in difficult matrices, which is why there is an increased activity in the last sampling time. An interesting study that may explain the adaptation of *L. plantarum* is proposed by Reveròn et al. [28], who propose a study of transcriptomics and the mechanism of action of *L. plantarum* in response to treatment with pure HT. *C. boidinii* strain used as a potentially resistant strain to several hurdles present in the matrix. Recently, De Melo Pereira et al. [29] reported that the genus *Candida* is commonly found in many fermented foods and beverages obtained by the main types of fermentation (alkaline, alcoholic, acetic, lactic, and mixed processes). In addition to its ubiquitous trait, the *Candida* genus also possesses a complex metabolic mechanism that allows it to survive, compete, and sometimes dominate fermentation processes [30]. Furthermore, it is known that a selected culture, besides the ability to control the fermentation process, should show the ability to survive in the fermentation environment and to exert acidifying activity through the production of organic acids. In the present study, results highlighted that *L. plantarum* inoculated samples exhibited the highest values of all detected acids. In a functional beverage, organic acids can play an additional role in protecting phenols, such as HT and TYR, from oxidation. In addition, different studies revealed that a lactic acid concentration of 0.5% (*v*/*v*) produced by LAB prevents pathogens’ growth, such as *Salmonella species, Escherichia coli*, and *Listeria monocytogenes* [31,32]. This result confirmed results previously reported, namely, that the fermentation driven by LAB leads to the production of mono–, di–, and tri–carboxylic acids, i.e., acetic, lactic, and propionic acids as intermediaries of biosynthetic metabolic pathways and amino acid metabolism. In detail, Okoye et al. [33] demonstrated through genome study that LAB contain unique and shared secondary metabolite biosynthetic gene clusters with bio preservative potential and a transcription factor, namely CRP (cyclic AMP receptor protein) endowed with novel binding sites involved in organic acid metabolism. 

Zooming in on biological activity, results obtained from tested microfiltered fermented OMWW and from pure HT, when tested at a 1:25 dilution, were found to be safe on chosen cell lines. In the present study, the choice of cell lines was based on taking into consideration that the HepG2 is one of the most reliable experimental models for prediction human liver toxicity. Indeed, the liver is responsible for most of the orally administered xenobiotic metabolism, for its anatomical proximity to the gastrointestinal tract and for its histological structure [34], whereas the Caco-2 cell line has been chosen as the most suitable in vitro model to rapidly assess the intestinal permeability and for xenobiotic transport studies [35,36]. Caco-2 cells exhibit a well–differentiated brush border on the apical surface and tight junctions, and express typical small–intestinal microvillus hydrolases and nutrient transporters. The crossing of biological membranes must be taken into account because it correlates with the ability of a pharmacologically active compound to reach the target site where performing the biological function. The intestinal transport of polyphenols seems to be strongly influenced by several factors such as food matrix, biotransformation and conjugation that occur during absorption [37,38]. Many studies have focused on the uptake of individual phenols, such as HT and TYR, which have shown good absorption across the cell membrane, while the uptake of a phytocomplex and how its different composition may affect the transport mechanism has been less explored [39]. In a recent study, Bartolomei et al. [40], demonstrated that a phenolic pool, extracted from extra virgin olive oil (EVOO), induced a protective effect against H_2_ O_2_-induced oxidative stress on Caco-2 and HepG2 cell lines. This observation demonstrated a selective transepithelial transport of certain oleuropein derivatives by Caco-2 cells, confirming that the phytocomplex could be transported with different mechanisms than those involved for single phenolic compounds, separately tested. According to results previously reported both phytocomplex composition and used starter cultures can significantly influence cell membrane crossing. In the present study, microbial cultures differently modulated the response of anti–inflammatory and antioxidant activity. It has been widely reported that phenols contained in EVOO reduce the reactive oxygen species (ROS) and malondialdehyde production, the nitric oxide release and the expression of inducible nitric oxide synthase (iNOS) and cyclooxygenase 2 (COX-2) [41]. Results obtained in the present study confirmed that OMWW samples affected the inhibitory activity towards COX-1 and COX-2, by a modulation of COX-2, according to previous in vitro reports on human monocytes [42]. The same authors demonstrated that HT attenuated ROS–mediated COX-2 transcription induced by bacterial lipopolysaccharide (LPS). COX catalyzes the first step in the biosynthesis of prostaglandins (PG), prostacyclin and thromboxane starting from free arachidonic acid (AA) [43]. Among prostaglandins, PGE_2_ is involved in inflammation, angiogenesis and in promoting the growth of several solid tumors, such as breast, ovarian, head and neck cancer, renal cell carcinoma and hematological cancers [44,45,46]. COX-1 and COX-2 are of great interest because they are targets of non–steroidal anti–inflammatory drugs (NSAIDs), which, when binding to the active site of COX, prevent the AA from reaching the catalytic pocket and, thus, the biosynthesis of prostaglandins. COX inhibition is therefore important in reducing the inflammatory response, tumorigenesis and cancer progression.

Many of the recognized anti–cancer properties of HT are related to other activities, such as ability to modulate the antioxidant system and ROS scavenge [47,48]. Ramirez–Tortosa et al. [49] demonstrated that a supplementation with HT (15 mg/day) is effective into downregulate several transcriptional factors, as described for other antioxidant agents, able to induce, at plasma level, a decrease of metalloproteinase in women with breast cancer. HT, as reported by the European Food Safety Authority but in general the phytocomplex present both in olive oil and in by–products, has a beneficial effect on human health. The interaction between phenols and microorganisms used as starters plays a key role in understanding the mechanism of action and how they can modulate the anti–inflammatory and antioxidant response in the development of degenerative diseases [50].

## 4. Materials and Methods

### 4.1. OMWW Sampling

The OMWW samples used in the present study were obtained by a three–phase olive oil extraction system at the Consoli oil company (Adrano, Italy) and collected during a two–year period. 

In detail, for Trial I OMWW was collected in the 2019–2020 season and for Trials II and III OMWW samples were collected in the 2020–2021 season. All the Trials are described in Figure 3. For Trial I the fresh produced OMWW was immediately stored at −20 °C at the Di3 A, University of Catania.

For Trial II, the OMWWs were stored at room temperature in the company facilities, until further treatments. To obtain a clear matrix, OMWW samples were subjected to filtration using Oenopad^®^ XF1, XF7 and XFSS filters (OENO S.R.L., Erbusco, Italy), suitable for food matrices and consisting of cellulose, diatomaceous earth and perlite. Different fractions were obtained: the as–is sample (prefiltered or PF sample); and the three fractions (F1, F2, F3) obtained by sequential filtration with filters at different porosity, as: the “XF1” filter (8.0–20 µm) to eliminate solid particulates; the”XF7” filter (2.0–4.0 µm) for clarifying step; the “XFSS” filter (0.20–0.40 µm) for final sterilization. All fractions were collected and stored at −20 °C.

In addition, the Trial III was obtained from the F3 sample, in turn obtained from Trial II, by microfiltration using the Sartoclear Dynamics^®^ kit (Sartorius, Varedo, Italy), connected to a vacuum pump. The latest process allows both the clarification/filtration and cold sterilization in a single step, as the used bottle presented a 0.22-µm polyethersulfone (PES) filter membrane. After processing samples needed for subsequent tests were frozen at −20 °C.

### 4.2. Set–Up of Fermentation Process

**Figure 4:**
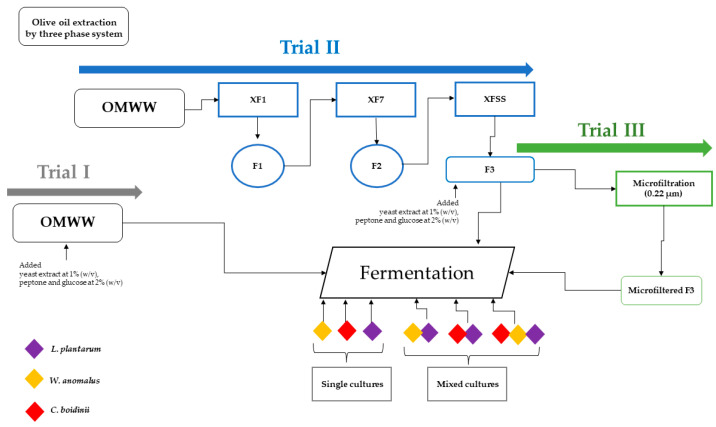
Fermentation process and OMWW obtained Trials.

In order to set up the fermentation process some components, such as yeast extract at 1% (*w*/*v*), peptone and glucose at 2% (*w*/*v*), were added into the fresh OMWW samples and to the F3 samples right before fermentation. All components were purchased from Liofilchem (Roseto degli Abruzzi, Italy). The fermentation process was started through the inoculum of microbial pools, consisting of yeast and lactic acid bacteria strains, belonging to the microbial culture collection of the Department of Agricultural, Food and Environmental Sciences (Di3 A) and to ProBioEtna srl, Spin off of University of Catania. In details, the *Candida boidinii* F3 30.1, *Wickerhamomyces anomalus* F5 60.5 and *Lactiplantibacillus plantarum* F 3.5 (DSM 34190) strains were used. All the strains were previously isolated from naturally fermented table olives [51]. One hundred microliters of each yeast inoculum and *L. plantarum* were spotted in Yeast Peptone Dextrose broth (YPD, Sigma-Aldrich, Milano, Italy) and de Man, Rogosa, and Sharpe broth (MRS, Oxoid, UK) and allowed to incubate overnight at a selective temperature of 25 °C and 32 °C, respectively. Then, the strains were inoculated at 0.5%, which corresponded to an initial cell density of 10^7^ CFU/mL for yeasts and 10^8^ CFU/mL for *L. plantarum* (Figure 4). Seven experimental samples were set up for each Trial: the un–inoculated samples (controls); three single culture inoculated samples; two samples inoculated with each yeast strain in mixed culture with the *L. plantarum* strain; one three–strain mixed culture sample. All tests were conducted in triplicate in an OMWW total volume of 400 mL. For Trial I and II, the fermentation process was monitored at regular intervals: at T0 (after about 7 h of microbial inoculation); T8 (after 8 days of fermentation); T30 (after 30 days of fermentation). For Trial III, fermentation parameters were monitored at T0, T8, T14 (after 14 days of fermentation) and T21 (end of fermentation). All fermentations were carried out at room temperature (20 ± 4 °C). 

### 4.3. Chemical Analyses

The pH, the TSS and the total phenol content were monitored for all samples during fermentation. The pH was measured with a Mettler DL25 pH meter (Mettler–Toledo International Inc., Columbus, OH, USA) and the total soluble solids (TSS), expressed as °Brix, were measured using a refractometer (Atago, RX-5000, Milano, Italy). In addition, the total phenolic content was determined according to the Folin–Ciocalteu’s colorimetric method (FC). The tested samples were mixed with 5 mL of commercial FC reagent (Labochimica, Campodarsego, Italy) diluted with water (1:10 *v*/*v*) and added with 4 mL of a 7.5% sodium carbonate solution. Subsequently, samples were left in the dark at room temperature. After 2 h, the absorbance was measured spectrophotometrically at 765 nm (Cary 100 Scan UV-Visible, Agilent, CA, USA). The total phenolic content was expressed as mg gallic acid equivalent (GAE)/L of sample).

### 4.4. Microbiological Analyses

Samples of Trial I, II and III were serially diluted and poured into agar plates containing specific media and incubated under specific conditions: de Man, Rogosa, and Sharpe Agar (MRSA, Oxoid, Milano, Italy) for lactic acid bacteria counts, incubated at 32 °C for 48 h under anaerobic conditions; Plate Count Agar (PCA, Oxoid, Milano, Italy) for mesophilic aerobic bacteria counts, incubated at 25 °C for 48 h; Violet Red Bile Glucose Agar (VRBGA, Liofilchem, Roseto degli Abruzzi, Italy IT) for the determination of Enterobacteriaceae, incubated aerobically at 30–35 °C for 18–24 h; Sabouraud Dextrose Agar (SAB, Bio-Rad, Hercules, CA, USA) for yeast counts, incubated at 25 °C for 48 h. At the end of fermentation, the presence/absence of *Clostridium perfringens* was also determined in Sulphite-Polymyxin-Sulphadiazine Agar (SPS, Oxoid, UK), incubated at 35–37 °C for 18–48 h, under anaerobic conditions. 

Moreover, for starter cultures monitoring, samples of Trial III were subjected to additional counting, in MRS agar and in SAB agar media, for *L. plantarum* and yeast determination, respectively.

All microbiological analyses were performed in triplicate and the results were expressed as Log CFU/mL.

### 4.5. HPLC Analysis

#### 4.5.1. Detection of Phenols

The HPLC analyses of fermented OMWW samples were performed by directly injecting the filtered samples (0.45 µm PTFE filters, Merck, Darmstadt, Germany) into the HPLC chromatographic system, i.e., Waters Alliance 2695 HPLC liquid chromatography equipped with a Waters 996 photodiode array (PDA) detector set at 280 nm and managed through the Waters Empower software (Waters Corporation, Milford, MA, USA). The column used was a Luna C18 (250 mm × 4.6 mm i.d., 5 m, 100 Å; Phenomenex, Torrance, CA, USA) maintained in an oven at 40 °C. A flow rate of 1 mL/min was used. Chromatographic separation was performed according to Romeo et al. [11]. The internal standard (I.S.), 50 mM pure gallic acid (Fluka, Buchs Switzerland), was used to quantify the phenolic compounds. The identification of phenolic compounds was obtained by comparing the peak retention time with those of pure standards of tyrosol (TYR), oleuropein (OLE), hydroxytyrosol (HT) chlorogenic acid, vanillic acid, caffeic acid, syringic acid, p-coumaric acid, ferulic acid, verbascoside, luteolin-7-o-glucoside, o-coumaric acid, rutin, oleuropein, apigenin-7-o-glucoside, luteolin-4-glucoside, quercetin, luteolin, apigenin (Extrasynthese, Genay, France). All analyses were performed in triplicate for each sample.

#### 4.5.2. Detection of Organic Acids

The determination of organic acids was carried out at the end of fermentation in Trial III. Each sample was filtered through a 0.45 μm PTFE syringe filter (Merck, Germany) before being injected into HPLC (HPLC instruments were described in the previous section) with a DAD detector set at 210 nm (and with spectrum acquisition from 200 to 400 nm). Isocratic elution with 5 mM sulfur acid was performed on a Rezex ROA Organic Acid H+ column (Phenomenex, CA, USA). The run time was set to 50 min at 0.6 mL/min. For calibration, pure standards of lactic, citric, acetic, propionic, isobutyric and butyric acids (all purchased from Sigma–Aldrich, Italy) were injected at different concentrations. All analyses were performed in triplicate for each sample.

### 4.6. Biological Assays

#### 4.6.1. Cell Culture and Cytotoxicity

Caco-2 cells were grown in Dulbecco’s Modified Eagle Medium high glucose (DMEM high glucose, Euroclone S.p.A., Pero, Italy) supplemented with 10% Fetal Bovine Serum (FBS, Euroclone S.p.A., Pero, Italy), 2 mM glutamine (Euroclone S.p.A., Pero, Italy), 100 U/mL of penicillin and 0.1 mg/mL of streptomycin (Euroclone). Caco-2 cells were kindly supplied from Dr. Aldo Cavallini and Dr. Caterina Messa from the Laboratory of Biochemistry National Institute for Digestive Diseases. “S. de Bellis”, Bari (Italy). 

Human hepatocellular liver carcinoma (HepG2) cell line was purchased from American Type Culture Collection (ATCC). HepG2 cells were cultured in Eagle’s Minimum Essential Medium (MEM, Euroclone), supplemented with 10% FBS, 2 mM glutamine (Euroclone), 100 U/mL penicillin and 0.1 mg/mL streptomycin (Euroclone S), 1% Non–Essential Amino Acids (NEAA, Euroclone). Cultured cells were maintained at 37 °C in atmosphere containing 95% of air and 5% of CO_2_. Cells were sub-cultivated every 48 h by trypsine–EDTA solution. 

Determination of cell growth was performed using the 3-(4.5-dimethylthiazol-2-yl)-2.5-diphenyltetrazolium bromide (MTT) assay (Sigma-Aldrich, Milan, Italy), 10.000 cells/well were seeded into 96-well plates at a volume of 100 µL. After 24 h, 100 µL of microfiltered fermented OMWW samples were added at the appropriate dilution: as such, 1:10, 1:25, 1:50 and 1:100 in triplicate. After 72 h incubation time with extracts, the plates containing the cells were incubated with MTT for 3–4 h at 37 °C and 5% of CO_2_. At the end of incubation time, MTT was aspirated, and the formazan crystals were solubilized by using 100 µL of dimethyl sulfoxide/ethanol (1:1) (Sigma–Aldrich). The absorbance values at λ = 570 nm were determined on the Victor Microplate Reader (PerkinElmer, Roma, Italy). Pure HT (Phytolab, Vastenbergsgreuth, Germany) was used as a positive control.

#### 4.6.2. Transport Caco-2 Monolayer

Caco-2 cells were seeded onto a Millicell-96 assay system (Millipore, Burlington, MA, USA) in which a cell monolayer was set in between a filter cell and a receiver plate at a density of 20.000 cells/well. The culture medium was replaced every 48 h and the cells were kept for 21 days in culture. The trans epithelial electrical resistance (TEER) of the monolayers was measured daily before and after the experiment by using an epithelial voltohmmeter (Millicell–ERS). Generally, TEER values greater than 1000 Ω for a 21-day culture are considered optimal. After 21 days of Caco-2 cell growth, the medium was removed from the filter wells and the receiver plate, and they were filled with fresh Hank’s balanced salt solution (HBSS) buffer (Invitrogen, Waltham, MA, USA). This procedure was repeated twice, and the plates were incubated at 37 °C for 30 min. After the incubation time, the HBSS buffer was removed and OMWW samples (dilution 1:100) were added to the filter well whereas fresh HBSS was added to the receiver plate. The plates were incubated at 37 °C for 120 min. Afterward, samples were removed from the apical (filter well) and basolateral (receiver plate) side of the monolayer to measure the permeability. The apparent permeability (P_app_) referred to HT in units of nm/second was calculated using the following Equation (1):

(1) $$P_{app}=\left(\frac{V_{A}}{Area\times time}\right)\times \left(\frac{\left[sample\right]acceptor}{\left[sample\right]initials}\right)$$

V_A_ = the volume (in mL) in the acceptor well; Area = the surface area of the membrane (0.11 cm^2^ of the well); time = the total transport time in seconds (7200 s); [sample]acceptor = the concentration of the sample measured by U.V. spectroscopy; [sample]initial = the initial sample concentration (1 × 10^−4^ M) in the apical or basolateral wells. 

#### 4.6.3. Cyclooxygenase Activity Inhibition

Preliminarily, the fermented OMWW samples obtained from Trial III were evaluated for their ability to inhibit *ovine*COX-1 or *human*COX-2 enzymes, measuring the extent (%) of enzyme activity inhibition at 50 µM, at dilution 1:25. The inhibition of the enzyme was evaluated by using a colorimetric COX inhibitor screening assay kit (Catalog No. 7601050, Cayman Chemicals, Ann Arbor, MI, USA) following the manufacturer’s instructions. COX is a bifunctional enzyme exhibiting both cyclooxygenase and peroxidase activities. The cyclooxygenase component catalyzes the conversion of arachidonic acid into the hydroperoxide PGG_2_ and then peroxidase component catalyzes PGG_2_ reduction into the corresponding alcohol PGH_2_, the precursor of PGs, thromboxane, and prostacyclin. The c COX inhibitor screening assay colorimetrically measures the peroxidase activity of the cyclooxygenases monitoring the appearance of oxidized N,N,N′,N′-tetramethyl-p-phenylenediamine (TMPD) at λ = 590 nm on the Victor Microplate Reader (PerkinElmer, Italy). Stock solutions of tested samples were dissolved in deionized distillated water.

#### 4.6.4. Antioxidant Activity 

Radical scavenging activity was determined as percentage of RSA (radical scavenging activity), according to Palmeri et al. [52]. The values were expressed using the following Equation (2):

(2) $$RSA\;\%=\left(\frac{\mathrm{Blank}\;\mathrm{Absorbance}\;-\;\mathrm{Sample}\;\mathrm{Absorbance}}{\mathrm{Blank}\;\mathrm{Absorbance}}\right)\times 100$$

Different dilutions of samples were added to the mixture of methanolic solution and 2,2-diphenyl-1-picrylhydrazyl radical 10^−4^ M. The DPPH absorbance values were evaluated at λ = 517 nm by monitoring the kinetics for 5 min with spectrophotometer (Shimadzu UV-1800, Denmark). Pure HT (Phytolab, Germany) was used as a positive control.

### 4.7. Statistical Analysis

Statistical analysis of the obtained results was performed by means of one–way analysis of variance (ANOVA) and Tukey’s HSD post hoc test for separation of means at a significance level of *p* ≤ 0.05. For data processing, SPSS software (version 21.0, IBM Statistics, NY, USA) was used for data processing.

## 5. Conclusions

The microfiltration process resulted in a suitable strategy to obtain a OMWW matrix able to be fermented. The use of selected microbial pools in single and co–cultures showed an increase in HT and TYR contents at the end of fermentation, compared with the control sample. Biological analyses showed that fermentation increases the antioxidant and inflammatory activity of OMWW that resulted to be safe in HepG2 and Caco-2 cell lines. In detail, the phenolic pattern associated to starter microorganisms exhibited an increase of active permeability on Caco-2 monolayer, and a moderate inhibition towards *o*COX-1 and *h*COX-2 was observed. The results confirm that fermented OMWW can be proposed as a new beverage and/or functional ingredient that could include the addition of compounds as flavorings and probiotic microorganisms. Despite the interesting results obtained at lab scale, perspective studies should aim to replay the process at the industrial scale to standardize phenol concentration at each obtained new formulation. 

## Figures and Tables

**Table 1:** Chemical parameters detected in samples of Trial III.

Sample	Time	pH	TSS (°Brix)	Total Phenol (mg/L)
Control	0	5.18 ± 0.01	8.30 ± 0.77	3627.4 ± 0.54 ^c^
*L. plantarum*	0	5.16 ± 0.01	10.60 ± 0.78	3711.2 ± 4.89 ^b^
*C. boidinii*	0	5.18 ± 0.01	10.28 ± 0.70	3539.8 ± 0.54 ^d^
*W. anomalus*	0	5.12 ± 0.08	9.76 ± 1.53	3172.9 ± 1.63 ^f^
*L.p + W.a*	0	5.13 ± 0.06	8.56 ± 0.80	4135.0 ± 4.89 ^a^
*L.p + C.b*	0	5.19 ± 0.02	10.04 ± 1.13	3474.7 ± 1.09 ^e^
*L.p + W.a + C.b*	0	5.18 ± 0.01	8.88 ± 1.39	2967.1 ± 2.18 ^g^
		n.s.	n.s.	**
Control	8	5.17 ± 0.01 ^a^	8.30 ± 0.78	1985.8 ± 3.26 ^g^
*L. plantarum*	8	5.04 ± 0.03 ^b^	10.30 ± 0.98	3032.5 ± 2.18 ^b^
*C. boidinii*	8	4.97 ± 0. 01 ^bcd^	8.88 ± 2.18	3020.6 ± 0.54 ^c^
*W. anomalus*	8	4.87 ± 0.02 ^e^	9.14 ± 1.77	2395.3 ± 1.63 ^f^
*L.p + W.a*	8	4.88 ± 0.04 ^de^	7.52 ± 1.17	2897.2 ± 0.01 ^e^
*L.p + C.b*	8	5.00 ± 0.01 ^bc^	9.50 ± 0.32	2991.0 ± 5.44 ^d^
*L.p + W.a + C.b*	8	4.94 ± 0.01 ^cde^	8.24 ± 1.29	3268.2 ± 1.63 ^a^
		**	n.s.	**
Control	14	5.18 ± 0.02 ^a^	8.30 ± 0.78	1809.5 ± 0.54 ^f^
*L. plantarum*	14	4.68 ± 0.01 ^cd^	9.90 ± 0.99	3282.9 ± 0.54 ^c^
*C. boidinii*	14	4.77 ± 0.01 ^b^	7.99 ± 2.82	2443.8 ± 1.63 ^d^
*W. anomalus*	14	4.67 ± 0.04 ^cd^	8.28 ± 1.44	3539.7 ± 0.54 ^b^
*L.p + W.a*	14	4.62 ± 0.09 ^d^	6.64 ± 1.52	2199.2 ± 3.81 ^e^
*L.p + C.b*	14	4.89 ± 0.01 ^b^	9.03 ± 1.12	3545.1 ± 53.84 ^b^
*L.p + W.a + C.b*	14	4.82 ± 0.02 ^bc^	7.62 ± 1.36	4015.0 ± 2.72 ^a^
		**	n.s.	**
Control	21	5.19 ± 0.01 ^a^	8.20 ± 0.61	1009.4 ± 0.54 ^f^
*L. plantarum*	21	4.65 ± 0.03 ^c^	8.15 ± 1.20	3392.1 ± 0.54 ^a^
*C. boidinii*	21	4.60 ± 0.01 ^d^	5.60 ± 0.57	3005.2 ± 0.54 ^b^
*W. anomalus*	21	4.54 ± 0.04 ^e^	6.36 ± 0.37	2394.2 ± 1.09 ^c^
*L.p + W.a*	21	4.49 ± 0.01 ^de^	5.32 ± 0.04	1914.6 ± 1.63 ^d^
*L.p + C.b*	21	4.84 ±0.02 ^b^	8.00 ± 1.41	3403.2 ± 10.88 ^a^
*L.p + W.a + C.b*	21	4.82 ± 0.01 ^b^	5.99 ± 0.01	1543.6 ± 1.09 ^e^
		**	n.s.	**

Data are expressed as mean ± standard deviations. Mean values with different letters within the same column at the same time interval are statistically different. n.s. not significant; ** Significance at *p* ≤ 0.01.

**Table 2:** Organic acids (mg/L) detected by HPLC.

Sample	Time (Days)	Citric Acid	Lactic Acid	Acetic Acid	Propionic Acid	Isobutyric Acid	Butyric Acid
Control	0	4172.9 ± 96.54	1606.6 ± 99.00	416.8 ± 97.31	3865.9 ± 268.47	3136.9 ± 188.31	0.00 ± 0.00
Control	21	4529.3 ± 100.00 ^de^	1219.2 ± 18.03 ^f^	326.3 ± 78.89 ^e^	3743.1 ± 34.21 ^g^	1654.9 ± 15.21 ^d^	566.4 ±48.79 ^d^
*L. plantarum*	21	7033.4 ± 15.76 ^a^	4512.6 ± 18.07 ^a^	7212.8 ± 82.59 ^a^	9802.4 ± 12.82 ^a^	3235.3 ± 5.51 ^a^	4666.4 ± 103.03 ^a^
*C. boidinii*	21	6624.4 ± 87.69 ^b^	4123.3 ± 20.03 ^b^	4568.4 ± 58.78 ^c^	9153.8 ± 19.41 ^b^	3202.7 ± 27.72 ^a^	4393.3 ± 44.23 ^a^
*W. anomalus*	21	5214.4 ± 121.00 ^c^	3774.5 ± 99.00 ^c^	6214.7 ± 168.83 ^b^	8219.2 ± 41.95 ^c^	2072.0 ± 77.52 ^b^	4239.4 ± 176.96 ^a^
*L.p + W.a*	21	4126.9 ± 106.79 ^f^	2846.0 ± 35.53 ^e^	6188.4 ± 85.52 ^b^	6831.2 ± 10.08 ^f^	1626.7 ± 1.41 ^e^	3682.8 ± 26.00 ^b^
*L.p + C.b*	21	4744.1 ± 16.31 ^d^	3167.5 ± 33.49 ^d^	4366.7 ± 132.82 ^c^	7913.0 ± 24.25 ^d^	2096.8 ± 16.35 ^b^	2995.9 ± 54.51 ^c^
*L.p + W.a + C.b*	21	4381.7 ± 20.88 ^ef^	3075.3 ± 31.95 ^d^	3334.0 ± 8.10 ^d^	7342.0 ± 116.15 ^e^	1810.2 ± 5.28 ^c^	3550.1 ± 25.58 ^b^

Data are expressed as mg/L of means ± standard deviations. Different letters indicate statistical differences within the same column (Significance at *p* ≤ 0.01).

**Table 3:** Apparent permeability of different samples of OMWW.

Samples	Concentration of HT (mg/L)	P_app_ BA (nm/s) Passive Transport	P_app_ AB (nm/s) Active Transport	BA/AB	λ (nm)	ε
Control	1.70	2581	457	4.22	275	0.80
*L. planturum*	9.50	4015	1014	3.95	285	0.20
*C. boidinii*	9.00	2540	575	4.41	275	0.09
*W. anomalus*	7.90	1958	367	5.34	285	0.22
*L.p + W.a*	8.00	1905	335	5.67	284	0.19
*L.p + C.b*	9.00	2912	1125	2.58	275	0.19
*L.p + W.a + C.b*	7.70	2587	522	4.95	283	0.20

All samples were tested at a dilution of 1:100. BA indicates basolateral to apical transport; AB indicates apical to basolateral transport; BA/AB values are from P_app_ AP–BL/P_app_ BL–AP.

## Supplementary Materials

The following supporting information can be downloaded at: https://www.mdpi.com/article/10.3390/molecules28020646/s1, Table S1. Chemical parameters detected in samples of Trial I and Trial II. Table S2. Main microbial groups counted in Trial I and Trial II samples during fermentation. Table S3. Evaluation of tested samples inhibition (as %) on COXs enzymes. Figure S1. Evaluation of inhibition (as %) of different HT concentrations on oCOX-1 and hCOX-2. Figure S2. (a) Trial I at the end of fermentation; (b) Cartons filters after the spinning process and samples from Trial II; (c) Sample of Trial II after the fermentation process. Figure S3. OMWW samples microfiltered at 0.22 µm.
