# Supplementary material for: Olive Mill Wastewater Fermented with Microbial Pools as a New Potential Functional Beverage
Source: Molecules. 2023 Jan 8;28(2):646. doi: 10.3390/molecules28020646 (PMC9866608; doi:10.3390/molecules28020646)
Supplement: Supplementary file 1 [file molecules-28-00646-s001.zip › molecules-2106830-supplementary.pdf]

**Table S1.** Chemical parameters detected in samples of Trial I and Trial II. **Table S2.** Main microbial groups counted in Trial I and Trial II samples during fermentation. **Table S3.** Evaluation of tested samples inhibition (as %) on COXs enzymes.
**Figure S1.** Evaluation of inhibition (as %) of different HT concentrations on oCOX-1 and hCOX-2. **Figure S2.** a) Trial I at the end of fermentation; b) Cartons filters after the spinning process and samples from trial II; c) Sample of Trial II after the fermentation process. **Figure S3.** OMWW samples microfiltered at 0.22 μm.

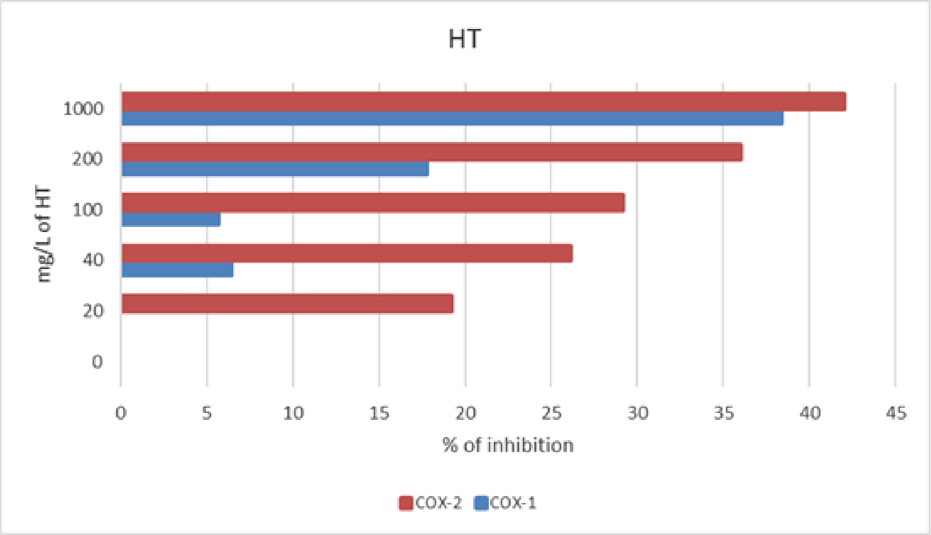


**Figure S1**

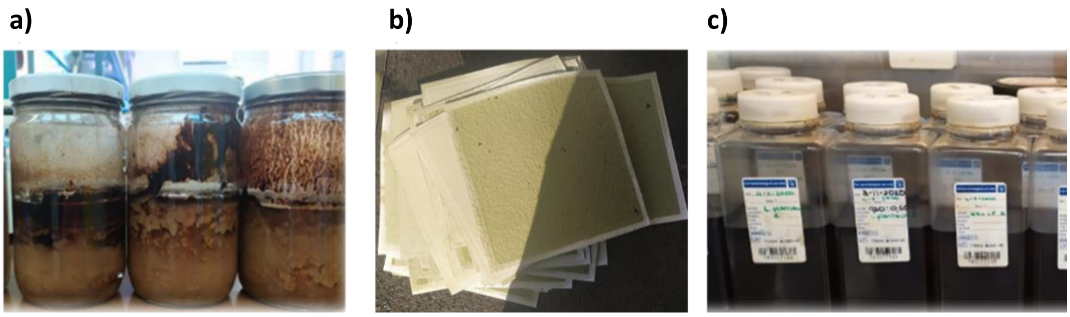


**Figure S2**

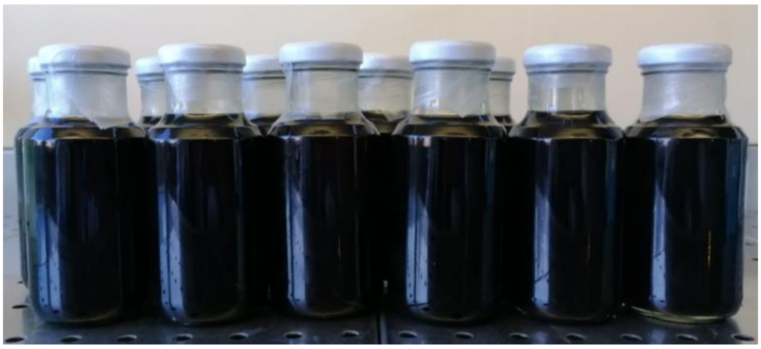

**Figure S3**

# Table S1

| Sample | Time (days) | pH | TSS (°Brix) | Total phenol (mg/L) | HT (mg/L) | TYR (mg/L) |
|---|---|---|---|---|---|---|
| **Trial I** | | | | | | |
| *Control* | 0 | 5.05 ± 0.01 | 7.77 ±0.02$^{b}$ | 2449.8 ± 0.25$^{d}$ | 410.1 ± 4.43$^{a}$ | 76.4 ± 0.46$^{a}$ |
| *L. plantarum* | 0 | 5.02 ± 0.01 | 7.72 ±0.01$^{b}$ | 2596.0 ± 1.42$^{b}$ | 285.4 ± 42.61$^{bc}$ | 68.2 ± 0.82$^{bc}$ |
| *C. boidinii* | 0 | 5.01 ± 0.01 | 7.73 ±0.08$^{b}$ | 2515.9 ± 0.15$^{c}$ | 415.2 ± 14.38$^{a}$ | 54.7 ± 1.49$^{d}$ |
| *W. anomalus* | 0 | 5.01 ± 0.01 | 8.21 ±0.02$^{a}$ | 2638.4 ± 0.59$^{b}$ | 373.9 ± 14.19$^{a}$ | 52.5 ± 3.28$^{d}$ |
| *L.p+ W.a* | 0 | 5.02 ± 0.02 | 7.08 ±0.01$^{c}$ | 2459.4 ± 0.79$^{c}$ | 345.2 ± 14.90$^{ab}$ | 61.5 ± 0.52$^{c}$ |
| *L.p+C.b* | 0 | 5.03 ± 0.01 | 8.32 ± 0.08$^{a}$ | 2654.7 ± 1.80$^{b}$ | 105.2 ± 2.66$^{d}$ | 54.3 ± 2.29$^{d}$ |
| *L.p+W.a+C.b* | 0 | 5.10 ± 0.14 | 8.19 ± 0.11$^{a}$ | 2744.9 ± 0.02$^{a}$ | 229.5 ± 6.62$^{c}$ | 74.6 ± 1.01$^{ab}$ |
| | | n.s | ** | ** | ** | ** |
| *Control* | 8 | 4.85 ± 0.01$^{a}$ | 6.27 ± 0.08$^{a}$ | 2037.5 ± 2.08$^{d}$ | 252.5 ± 1.05$^{b}$ | 89.8 ± 6.42 |
| *L. plantarum* | 8 | 4.76 ± 0.03$^{ab}$ | 5.65 ± 0.06$^{b}$ | 1584.7 ± 0.41$^{e}$ | 174.6 ± 2.06$^{d}$ | 88.8 ± 3.14 |
| *C. boidinii* | 8 | 4.45 ± 0.07$^{c}$ | 5.79 ± 0.02$^{b}$ | 2893.4 ± 7.62$^{a}$ | 305.2 ± 14.17$^{a}$ | 98.2 ± 7.38 |
| *W. anomalus* | 8 | 4.82 ± 0.03$^{a}$ | 5.73 ± 0.09$^{b}$ | 2336.3 ± 0.41$^{b}$ | 224.4 ± 0.93$^{c}$ | 113.1 ± 16.44 |
| *L.p+ W.a* | 8 | 4.69 ± 0.01$^{b}$ | 5.19 ±0.01$^{c}$ | 2363.6 ± 3.39$^{b}$ | 267.9 ± 2.20$^{b}$ | 112.7 ± 4.63 |
| *L.p+C.b* | 8 | 4.76 ± 0.01$^{ab}$ | 6.19 ±0.05$^{a}$ | 2047.2 ± 6.69$^{d}$ | 266.8 ± 3.65$^{b}$ | 103.2 ± 1.76 |
| *L.p+W.a+C.b* | 8 | 4.64 ± 0.01$^{b}$ | 6.16 ± 0.08$^{a}$ | 2191.8 ± 2.49$^{c}$ | 300.0 ± 3.08$^{a}$ | 108.9 ± 2.12 |
| | | ** | ** | *** | * | n.s |
| *Control* | 30 | 4. 99 ± 0.02$^{b}$ | 6.31 ± 0.14$^{a}$ | 2642.5 ± 1.30$^{b}$ | 1283.6 ± 23.21$^{c}$ | 439.8 ± 67.33$^{b}$ |
| *L. plantarum* | 30 | 5.51 ± 0.14$^{a}$ | 5.99 ± 0.01$^{ab}$ | 2485.1 ± 12.90$^{c}$ | 1516.6 ± 153.76$^{bc}$ | 511.5 ± 40.51$^{ab}$ |
| *C. boidinii* | 30 | 4.81 ± 0.01$^{bc}$ | 5.60 ± 0.28$^{b}$ | 3135.5 ± 5.23$^{ab}$ | 2190.2 ± 155.64$^{ab}$ | 679.4 ± 9.71$^{a}$ |
| *W. anomalus* | 30 | 4.59 ± 0.01$^{cd}$ | 5.95 ± 0.08$^{ab}$ | 3241.9 ± 0.13$^{a}$ | 2630.4 ± 44.05$^{a}$ | 537.8 ± 9.71$^{ab}$ |
| *L.p+ W.a* | 30 | 4.55 ± 0.07$^{d}$ | 6.07 ± 0.09$^{ab}$ | 2555.9 ± 4.30$^{b}$ | 1622.1 ± 80.47$^{bc}$ | 529.0 ± 12.03$^{ab}$ |
| *L.p+C.b* | 30 | 4.71 ± 0.01$^{cd}$ | 6.05 ± 0.07$^{ab}$ | 2335.6 ± 5.34$^{d}$ | 1560.0 ± 80.26$^{bc}$ | 508.6 ± 5.84$^{ab}$ |
| *L.p+W.a+C.b* | 30 | 4.63 ± 0.05c$^{d}$ | 6.34 ± 0.01$^{a}$ | 3129.7 ± 4.63$^{ab}$ | 1543.3 ± 244.36$^{bc}$ | 539.4 ± 3.24$^{ab}$ |
| | | ** | * | ** | * | * |
| **Trial II** | | | | | | |
| *Control* | 0 | 5.36 ± 0.02$^{ab}$ | 7.88 ± 0.16$^{bc}$ | 3773.7 ± 7.26$^{a}$ | 727.6 ± 39.38$^{bc}$ | 382.5 ± 3.54$^{b}$ |
| *L. plantarum* | 0 | 5.30 ± 0.14$^{ab}$ | 7.67 ± 0.06$^{c}$ | 3655.2 ± 0.55$^{b}$ | 479.3 ± 31.42$^{d}$ | 262.0 ± 7.25$^{cd}$ |
| *C. boidinii* | 0 | 5.30 ± 0.01$^{ab}$ | 8.05 ± 0.07$^{b}$ | 3271.4 ± 14.62$^{c}$ | 616.6 ± 60.40$^{c}$ | 245.4 ± 12.34$^{d}$ |
| *W. anomalus* | 0 | 5.47 ± 0.03$^{a}$ | 8.01 ± 0.01$^{b}$ | 3778.9 ± 12.59$^{a}$ | 402.8 ± 0.08$^{d}$ | 528.4 ± 0.39$^{a}$ |
| *L.p+ W.a* | 0 | 5.20 ± 0.01$^{b}$ | 7.84 ± 0.08$^{bc}$ | 3653.1 ± 5.48$^{b}$ | 802.3 ± 12.26$^{ab}$ | 332.8 ± 47.82$^{bc}$ |
| *L.p+C.b* | 0 | 5.37 ± 0.01$^{ab}$ | 8.56 ± 0.01$^{a}$ | 2957.8 ± 10.03$^{e}$ | 874.3 ± 3.34$^{a}$ | 245.1 ± 10.16$^{d}$ |
| *L.p+W.a+C.b* | 0 | 5.32 ± 0.02$^{ab}$ | 8.06 ± 0.08$^{b}$ | 3077.2 ± 1.13$^{d}$ | 765.9 ± 33.78$^{ab}$ | 299.8 ± 5.06$^{cd}$ |
| | | * | ** | ** | ** | ** |
| *Control* | 8 | 4.35 ± 0.07$^{a}$ | 6.47 ± 0.04$^{a}$ | 2992.6 ± 1.85$^{d}$ | 526.2 ± 4.64$^{bcd}$ | 166.2 ± 3.79$^{c}$ |
| *L. plantarum* | 8 | 3.97 ± 0.01$^{c}$ | 5.64 ± 0.06$^{e}$ | 3005.1 ± 7.29$^{c}$ | 594.7 ± 0.49$^{bc}$ | 211.2 ± 22.84$^{b}$ |
| *C. boidinii* | 8 | 4.05 ± 0.01$^{c}$ | 6.05 ± 0.07$^{cd}$ | 3201.8 ± 1.12$^{d}$ | 306.1 ± 0.16$^{d}$ | 120.0 ± 0.05$^{d}$ |
| *W. anomalus* | 8 | 3.97 ± 0.01$^{c}$ | 5.89 ± 0.01$^{d}$ | 3261.9 ± 4.15$^{b}$ | 393.2 ± 5.78$^{cd}$ | 121.7 ± 1.11$^{d}$ |
| *L.p+ W.a* | 8 | 4.18 ± 0.01$^{b}$ | 5.50 ± 0.01$^{e}$ | 3200.7 ± 3.78$^{d}$ | 1064.9 ± 15.22$^{a}$ | 280.8 ± 4.01$^{a}$ |
| *L.p+C.b* | 8 | 3.98 ± 0.01$^{c}$ | 6.16 ± 0.08$^{bc}$ | 3024.1 ± 28.40$^{c}$ | 314.2 ±146.37$^{d}$ | 171.8 ± 4.39$^{c}$ |
| *L.p+W.a+C.b* | 8 | 3.99 ± 0.01$^{c}$ | 6.36 ± 0.05$^{ab}$ | 3379.5 ± 26.15$^{a}$ | 666.9 ± 12.83$^{b}$ | 142.8 ± 3.70$^{cd}$ |
| | | ** | ** | ** | ** | ** |
| *Control* | 30 | 6.29 ± 0.01$^{a}$ | 6.50 ± 0.01$^{a}$ | 2796.7 ± 1.85$^{e}$ | 330.7 ± 2.17$^{d}$ | 129.5 ± 0.34$^{b}$ |
| *L. plantarum* | 30 | 4.15 ± 0.07$^{e}$ | 5.64 ± 0.02$^{c}$ | 3577.6 ± 12.40$^{a}$ | 840.3 ± 6.68$^{b}$ | 126.0 ± 1.38$^{b}$ |
| *C. boidinii* | 30 | 5.57 ± 0.01$^{b}$ | 5.84 ± 0.06$^{bc}$ | 3267.2 ± 2.28$^{c}$ | 979.2 ± 8.26$^{b}$ | 87.0 ± 0.27$^{cd}$ |
| *W. anomalus* | 30 | 5.64 ± 0.06$^{b}$ | 5.94 ± 0.08$^{b}$ | 3160.4 ± 41.87$^{d}$ | 596.3 ± 89.00$^{c}$ | 141.4 ± 1.63$^{b}$ |
| *L.p+ W.a* | 30 | 5.04 ± 0.06$^{c}$ | 5.68 ± 0.11$^{c}$ | 3364.1 ± 25.20$^{bc}$ | 1235.6 ± 38.93$^{a}$ | 306.2 ± 12.98$^{a}$ |
| *L.p+C.b* | 30 | 4.54 ± 0.06$^{d}$ | 6.05 ± 0.07$^{b}$ | 3426.8 ± 16.75$^{b}$ | 810.7 ± 87.83$^{b}$ | 67.5 ± 0.85$^{d}$ |
| *L.p+W.a+C.b* | 30 | 5.59 ± 0.01$^{b}$ | 6.36 ± 0.01$^{a}$ | 3348.4 ± 39.05$^{bc}$ | 827.2 ± 12.99$^{b}$ | 100.3 ± 0.11$^{c}$ |
| | | ** | ** | ** | ** | ** |

Data are expressed as Log CFU/mL mean ± standard deviations. Mean values with different letters within the same column at the same time interval are statistically different. N.s. not significant; *Significance at $P < 0.05$; **Significance at $P < 0.01$.

## Table S2

| Sample | Time (days) | LAB | Yeasts and moulds | Aerobic mesophilic bacteria | Enterobacteriaceae | Staphylococci |
|---|---|---|---|---|---|---|
| **Trial I** | | | | | | |
| *Control* | 0 | 5.23 ± 0.01$^{d}$ | 6.50 ± 0.04$^{b}$ | 6.29 ± 0.16$^{d}$ | 6.90 ± 0.01$^{a}$ | 1.06 ± 0.03$^{e}$ |
| *L. plantarum* | 0 | 5.85 ± 0.02$^{a}$ | 5.69 ± 0.01$^{e}$ | 7.36 ± 0.03$^{a}$ | 6.94 ± 0.01$^{a}$ | 1.06± 0.03$^{e}$ |
| *C. boidinii* | 0 | 5.31 ± 0.01$^{c}$ | 5.49 ± 0.01$^{f}$ | 5.96 ± 0.01$^{e}$ | 6.79 ± 0.01$^{b}$ | 3.98 ± 0.03$^{b}$ |
| *W. anomalus* | 0 | 4.78 ± 0.04$^{f}$ | 5.48 ± 0.01$^{f}$ | 7.06 ± 0.08$^{b}$ | 6.57 ± 0.03$^{c}$ | 2.63 ± 0.21$^{d}$ |
| *L.p+W.a* | 0 | 5.48 ± 0.02$^{b}$ | 6.19 ± 0.02$^{d}$ | 6.85 ± 0.01$^{bc}$ | 6.33 ± 0.01$^{d}$ | 4.79 ± 0.01$^{a}$ |
| *L.p+C.b* | 0 | 5.30 ± 0.04$^{c}$ | 6.96 ± 0.01$^{a}$ | 6.72 ± 0.03$^{c}$ | 6.36 ± 0.02$^{d}$ | 3.30 ± 0.01*c* |
| *L.p+W.a+C.b* | 0 | 5.15 ± 0.12$^{e}$ | 6.28 ± 0.03$^{c}$ | 7.50 ± 0.04$^{a}$ | 6.57 ± 0.01$^{c}$ | 4.59± 0.16$^{a}$ |
| | | ** | ** | ** | ** | ** |
| *Control* | 8 | 6.74 ± 0.03$^{a}$ | 8.15 ± 0.21$^{a}$ | 8.15 ± 0.21$^{a}$ | 5.00± 0.01$^{bc}$ | 1.63± 0.40 |
| *L. plantarum* | 8 | 6.60 ± 0.01$^{c}$ | 6.88 ± 0.04$^{b}$ | 6.83 ± 0.$^{01b}$ | 4.61± 0.01$^{c}$ | 3.30 ± 0.43 |
| *C. boidinii* | 8 | 6.59 ± 0.01$^{c}$ | 7.06 ± 0.05$^{b}$ | 6.43 ± 0.07$^{b}$ | 0.00± 0.01$^{d}$ | 4.29± 0.01 |
| *W. anomalus* | 8 | 6.37 ± 0.02$^{e}$ | 6.85 ± 0.01$^{b}$ | 6.77 ± 0.01$^{c}$ | 6.02± 0.03$^{ab}$ | 3.00 ± 0.41 |
| *L.p+W.a* | 8 | 6.74 ± 0.05$^{a}$ | 7.28 ± 0.16$^{b}$ | 5.08 ± 0.01$^{d}$ | 4.85± 0.01$^{c}$ | 3.88 ± 0.03 |
| *L.p+C.b* | 8 | 6.62 ± 0.08$^{b}$ | 7.28 ± 0.31$^{b}$ | 6.57 ± 0.01$^{bc}$ | 5.34± 0.06$^{abc}$ | 4.37 ± 0.27 |
| *L.p+W.a+C.b* | 8 | 6.48 ± 0.01$^{d}$ | 6.98 ± 0.01$^{b}$ | 5.35 ± 0.01$^{d}$ | 6.17± 0.74*a* | 4.12± 0.07 |
| | | ** | * | ** | ** | n.s |
| *Control* | 30 | 6.43± 0.01$^{a}$ | 6.56 ± 0.17$^{b}$ | 6.07 ± 0.01$^{c}$ | 2.23 ± 0.34$^{a}$ | 3.05 ± 0.06$^{a}$ |
| *L. plantarum* | 30 | 5.32± 0.05$^{bc}$ | 6.35 ± 0.15b$^{c}$ | 7.00 ± 0.01$^{a}$ | 0.00 ± 0.00$^{b}$ | 0.00 ± 0.00$^{b}$ |
| *C. boidinii* | 30 | 4.78 ± 0.06$^{c}$ | 6.04 ± 0.15b$^{c}$ | 5.72 ± 0.03$^{d}$ | 0.00 ± 0.00$^{b}$ | 0.00 ± 0.00$^{b}$ |
| *W. anomalus* | 30 | 6.89 ± 0.09$^{a}$ | 6.33 ± 0.01b$^{c}$ | 6.23 ± 0.02$^{c}$ | 0.00 ± 0.00$^{b}$ | 0.00 ± 0.00$^{b}$ |
| *L.p+W.a* | 30 | 6.05 ±0.02$^{ab}$ | 5.77 ± 0.10$^{d}$ | 6.05 ± 0.08$^{c}$ | 0.00 ± 0.00$^{b}$ | 0.00 ± 0.00$^{b}$ |
| *L.p+C.b* | 30 | 6.44 ± 0.04$^{a}$ | 6.41 ± 0.08$^{bc}$ | 6.07 ± 0.10$^{c}$ | 0.00 ± 0.00$^{b}$ | 0.00 ± 0.00$^{b}$ |
| *L.p+W.a+C.b* | 30 | 7.03 ± 0.01$^{a}$ | 7.71 ± 0.01$^{a}$ | 6.64 ± 0.01$^{b}$ | 0.00 ± 0.00$^{b}$ | 0.00 ± 0.00$^{b}$ |
| | | * | ** | ** | ** | ** |
| **Trial II** | | | | | | |
| *Control* | 0 | 1.30 ± 0.01$^{e}$ | 2.48 ± 0.01$^{c}$ | 5.38 ± 0.55$^{bc}$ | 0.00 ± 0.00$^{d}$ | 0.00 ± 0.00$^{f}$ |
| *L. plantarum* | 0 | 5.80 ± 0.17$^{a}$ | 5.41 ± 0.12$^{ab}$ | 4.15± 0.21$^{e}$ | 2.14 ± 0.09$^{ab}$ | 1.06 ± 0.03$^{e}$ |
| *C. boidinii* | 0 | 5.75 ± 0.03$^{a}$ | 5.51 ± 0.77$^{ab}$ | 4.23 ± 0.34$^{de}$ | 2.88 ± 0.02 $^{a}$ | 3.97 ± 0.03$^{b}$ |
| *W. anomalus* | 0 | 2.00 ± 0.01$^{d}$ | 4.82 ± 0.01$^{ab}$ | 5.26 ± 0.38$^{cde}$ | 1.09 ± 0.12c | 2.65 ± 0.21$^{d}$ |
| *L.p+ W.a* | 0 | 4.77 ± 0.01$^{c}$ | 4.64 ± 0.29$^{ab}$ | 5.65 ± 0.11$^{ab}$ | 2.77 ± 0.49 $^{a}$ | 4.79 ± 0.01$^{a}$ |
| *L.p.+C.b* | 0 | 5.00 ± 0.00$^{bc}$ | 4.20 ± 0.04$^{c}$ | 7.62 ± 0.04$^{a}$ | 1.60 ± 0.09$^{bc}$ | 3.30 ± 0.01$^{c}$ |
| *L.p+W.a+C.b* | 0 | 5.31 ± 0.20$^{b}$ | 5.58 ± 0.19$^{a}$ | 6.71 ± 0.02$^{ab}$ | 1.02 ± 0.03$^{c}$ | 4.84 ± 0.28$^{a}$ |
| | | * | ** | ** | ** | ** |
| *Control* | 8 | 5.43 ± 0.45$^{b}$ | 5.92 ± 0.04$^{b}$ | 7.38 ± 0.55$^{a}$ | 5.26 ± 0.58 | 4.60 ± 0.16$^{b}$ |
| *L. plantarum* | 8 | 7.91 ± 0.01$^{a}$ | 7.84 ± 0.09$^{a}$ | 5.82 ± 0.18$^{b}$ | 4.17 ± 0.21 | 0.00 ± 0.00$^{d}$ |
| *C. boidinii* | 8 | 7.50 ± 0.04 $^{a}$ | 7.61 ± 0.01$^{a}$ | 7.36 ± 0.51$^{a}$ | 4.20 ± 0.24 | 3.00 ± 0.00$^{c}$ |
| *W. anomalus* | 8 | 7.91 ± 0.01 $^{a}$ | 7.50 ± 0.65$^{a}$ | 7.84 ± 0.09$^{a}$ | 4.39 ± 0.43 | 3.39 ± 0.43$^{c}$ |
| *L.p+W.a* | 8 | 7.58 ± 0.17 $^{a}$ | 7.47 ± 0.49$^{a}$ | 7.49 ± 0.02$^{a}$ | 4.38 ± 0.41 | 4.38 ± 0.41$^{b}$ |
| *L.p.+C.b* | 8 | 7.62 ± 0.01 $^{a}$ | 7.00 ± 0.21$^{ab}$ | 7.00 ± 0.01$^{a}$ | 4.60 ± 0.01 | 3.90 ± 0.03$^{bc}$ |
| *L.p+W.a+C.b* | 8 | 7.61 ± 0.02 $^{a}$ | 7.85 ± 0.19$^{a}$ | 5.30 ± 0.01$^{b}$ | 4.04 ± 0.06 | 5.72 ± 0.03$^{a}$ |
| | | ** | * | ** | n.s | ** |
| *Control* | 30 | 7.77 ± 0.01 $^{a}$ | 7.84 ± 0.01$^{d}$ | 7.75 ± 0.01$^{b}$ | 0.00 ± 0.00 | 0.00 ± 0.00 |
| *L. plantarum* | 30 | 7.36 ± 0.08 $^{a}$ | 7.29 ± 0.02$^{f}$ | 7.30 ± 0.01$^{c}$ | 0.00 ± 0.00 | 0.00 ± 0.00 |
| *C. boidinii* | 30 | 7.72 ± 0.03 $^{a}$ | 8.00 ± 0.01$^{c}$ | 7.31 ± 0.01$^{c}$ | 0.00 ± 0.00 | 0.00 ± 0.00 |
| *W. anomalus* | 30 | 5.00 ±0.01$^{b}$ | 6.00 ± 0.01$^{g}$ | 7.86 ± 0.03$^{a}$ | 0.00 ± 0.00 | 0.00 ± 0.00 |
| *L.p+W.a* | 30 | 8.44 ± 0.66 $^{a}$ | 8.77 ± 0.02$^{b}$ | 7.30 ± 0.01$^{c}$ | 0.00 ± 0.00 | 0.00 ± 0.00 |
| *L.p+C.b* | 30 | 7.80 ± 0.04 $^{a}$ | 9.00 ± 0.01$^{a}$ | 7.86 ± 0.01 $^{a}$ | 0.00 ± 0.00 | 0.00 ± 0.00 |
| *L.p+W.a+C.b* | 30 | 7.30 ± 0.01 $^{a}$ | 7.43 ± 0.03$^{e}$ | 7.31 ± 0.03$^{c}$ | 0.00 ± 0.00 | 0.00 ± 0.00 |
| | | ** | ** | ** | n.s | n.s |

Data are expressed as Log CFU/mL mean ± standard deviations. Mean values with different letters within the same column at the same time interval are statistically different. N.s. not significant; *Significance at $P < 0.05$; **Significance at $P < 0.01$.

## Table S3

| **Samples** | **Concentration of HT (mg/L)** | **Inhibition *o*COX-1 (%)** | **Inhibition *h*COX-2 (%)** |
|---|---|---|---|
| *Control* | 6.80 | 0.00 | 0.00 |
| *L.planturum* | 37.02 | 5 .09 | 0.00 |
| *C. boidinii* | 36.09 | 15.96 | 12.95 |
| *W. anomalus* | 31.72 | 1.32 | 3.27 |
| *L.p + W.a* | 32.06 | 0.19 | 0.86 |
| *L.p + C.b* | 36.13 | 8.20 | 0.00 |
| *L.p + W.a + C.b* | 30.71 | 0.00 | 5.78 |
